# The inverse relation between changes in body weight and serum phosphate expresses weight loss after lifestyle intervention in non-smokers and smokers, but not in subjects who quit smoking

**DOI:** 10.3389/fnut.2025.1741580

**Published:** 2026-01-15

**Authors:** Sofia Håglin, Lennart Bäckman, Lena Håglin

**Affiliations:** Department of Public Health and Clinical Medicine, Family Medicine, Umeå University, Umeå, Sweden

**Keywords:** intervention program, obesity, phosphate, smoking cessation, triglycerides, weight change

## Abstract

**Background/objectives:**

Metabolic alterations, physical activity, and dietary pattern together can explain why smoking cessation (SC) often results in weight gain. We studied changes in weight and changes in cardiovascular (CVD) risk factors after an intervention that included an exercise program, dietary advice, and stress management.

**Subjects/methods:**

The patient population (*n* = 1,782) attended the Vindeln Patient Education Centre (VPE-center) for a 4-week comprehensive lifestyle intervention program, which included the option of smoking cessation. The data were collected before and after, at either the 6- or 12-month follow-up on 247 smokers, 95 former smokers, and 1,440 non-smokers.

**Results:**

A high CVD-risk population lost weight and had decreased serum triglyceride (S-TG) and increased serum phosphate (S-P) at the 6- or 12-month follow-up. At baseline, smokers and former smokers had higher S-TG and lower systolic blood pressure (SBP) than non-smokers. Smokers had higher S-P at baseline and higher S-TG at follow-up than non-smokers. A daily energy intake of around 6,300 kJ (1,500 kcal) and a schedule of physical activity resulted in weight loss, but to a significantly lesser extent in patients who quit smoking compared to patients who continued to smoke or stayed as non-smokers. With SC, a decrease in S-P was associated with weight gain, whereas an increase in S-P was associated with weight loss.

**Conclusion:**

An interaction between smoking habits, body mass index (BMI), and S-P may express a metabolic change that contributes to the degree of weight loss. Differences in changed metabolic response according to smoking habits express effects from smoking cessation, besides differences in the amount of weight change.

## Introduction

Managing early post-smoking cessation (post-SC) weight gain can limit the risk for cardiovascular disease (1), type-2 diabetes (2, 3), and metabolic syndrome (4, 5). The critical condition is associated with a large and early increase in weight after quitting (2, 6), not necessarily further weight gain in the long run (7, 8). Increased body weight gives rise to higher blood lipids and blood pressure (9, 10), which may diminish the health benefits intended by SC. Nonetheless, a decreased risk of coronary heart disease was reported to be associated with weight gain (10). Differences in outcomes across studies on health and disease prevention from SC may be associated with time to follow-up, population size, predictors, and patterns of body weight gain.

The degree of weight gain in connection with SC seems to depend on body mass index (BMI) before cessation (11) and is not only a result of post-cessation changes in dietary pattern or physical activity (12, 13). A fourfold increase in odds of gaining more than 13 kg was associated with underweight females (14), shown also for low tobacco consumption and low BMI (15). The large weight gain early after cessation indicates that some metabolic changes are triggered by SC, whereas long-term weight gain may result from changes in habits and lifestyle patterns.

A multidisciplinary approach within medical care limits weight gain associated with SC (16–18) by providing early support and advice on weight control. A structured program of dietary advice with focus on weight control (19), energy intake (20), and physical activity (21) and changing health behaviors (12) might attract smokers who fear weight gain. A combination of pharmaceutical effects and adaptation to a negative energy balance by lower food intake and higher activity may be the most optimal strategies for SC programs (22).

Here, we studied the association between weight changes and obesity related disturbance linked to cardiovascular disease (CVD) risk after an intervention including an exercise program, dietary advice, and stress management according to smoking habits.

## Subjects and methods

### Recruitment of patients

This intervention study took place between 1984 and 1997, when primary health care physicians in Västerbotten referred people at high CVD risk to Vindeln Patient Education Centre (VPE-center). Eight new groups of patients were admitted each year, and 29 groups of patients were admitted with follow-ups after 6 months. Between June 1987 and April 1997, 80 groups were admitted with planned follow-ups after 12 months. The total cohort of 2,504 patients (1,096 males and 1,408 females) had at least one risk factor for CVD, such as hypertension or diabetes, in addition to being overweight or obese (23).

About 30 patients were admitted at a time, and they spent 4 weeks at the center, with interruptions on weekends. On admission, patients underwent clinical and physical examinations to confirm the referring physician’s diagnosis. A journal was set up for each patient and all data, including for the follow-up periods, were collected at the center. In the journal, body mass index (BMI) (kg/m^2^), systolic blood pressure (SBP) (mm Hg), and diastolic blood pressure (DBP) (mm Hg), measurements obtained in a sitting position were recorded. Blood pressure, blood lipids, and serum phosphate (S-P), urate (S-Urate), and gamma-glutamyl-transferase (S-GGT) were also measured at baseline and at the 6-month or 12-month follow-up. The patient population was followed for either 6 months or 12 months. The time of decision to quit or continue smoking was recorded during the 4 weeks of the intervention program and confirmed at the time of follow-up. Characteristics of the studied population at admittance to the VPE-center and at follow-up are presented in Table 1. The data was collected for 247 smokers, 95 former smokers, and 1,440 non-smokers.

**Table 1:** Differences in metabolic risk factors among smokers (1), non-smokers (3), and those who quit smoking (2) (mean ± SD) at baseline and at follow-up.

Variables	Current Smokers (1)	Former smokers (2)	Non-smokers (3)	Group test	*p*
Age, years *n*	47.1 ± 9.7 247	48.6 ± 9.4 95	51.8 ± 9.5 1,440	1–2 1–3 2–3	0.593 **<0.001** **0.005**
T2DM, % *n*	17.0 42/247	17.9 17/95	18.8 270/1,440	1–2 1–3 2–3	0.845 0.514 0.836
Sex, male % *n* (males/females)	44.5 110/137	50.5 48/47	42.8 617/823	1–2 1–3 2–3	0.319 0.621 0.143
BMI, kg/m^2^					
Baseline *n*	31.0 ± 5.5 247	29.8 ± 5.1 95	31.0 ± 5.3 1,440	1–2 1–3 2–3	0.199 1.000 0.101
Follow-up *n*	29.4 ± 5.2 241	29.4 ± 5.2 95	29.5 ± 5.0 1,414	1–2 1–3 2–3	1.000 1.000 1.000
Change *n*	−1.66 ± 2.27 241	−0.52 ± 1.84 92	−1.54 ± 1.84 1,414	1–2 1–3 2–3	**<0.001** 1.000 **<0.001**
SBP, mmHg					
Baseline *n*	143 ± 19 247	143 ± 17 95	148 ± 19 1,440	1–2 1–3 2–3	1.000 **<0.001** **0.010**
Follow-up *n*	137 ± 17 245	138 ± 16 95	141 ± 17 1,427	1–2 1–3 2–3	1.000 **0.003** 0.351
Change *n*	−6.4 ± 17.5 245	−4.7 ± 20.5 95	−7.9 ± 18.7 1,427	1–2 1–3 2–3	1.000 0.748 0.347
DBP, mmHg					
Baseline *n*	86 ± 12 246	86 ± 11 95	89 ± 11 1,421	1–2 1–3 2–3	1.000 **<0.001** 0.053
Follow-up *n*	82 ± 10 245	83 ± 10 95	84 ± 10 1,431	1–2 1–3 2–3	0.733 0.051 1.000
Change *n*	−4.3 ± 10.9 244	−2.9 ± 12.6 95	−5.6 ± 11.6 1,414	1–2 1–3 2–3	1.000 0.299 0.092
S-Chol, mmol/L					
Baseline *n*	6.65 ± 1.43 247	6.55 ± 1.31 95	6.67 ± 1.39 1,440	1–2 1–3 2–3	1.000 1.000 1.000
Follow-up *n*	6.58 ± 1.28 220	6.46 ± 1.54 88	6.51 ± 1.33 1,347	1–2 1–3 2–3	1.000 1.000 1.000
Change *n*	−0.13 ± 1.01 220	−0.04 ± 1.08 88	−0.17 ± 1.00 1,347	1–2 1–3 2–3	1.000 1.000 0.765
S-TG, mmol/L					
Baseline *n*	2.81 ± 2.48 247	2.83 ± 2.27 95	2.34 ± 1.52 1,440	1–2 1–3 2–3	1.000 **<0.001** **0.018**
Follow-up *n*	2.42 ± 1.58 218	2.39 ± 1.77 88	2.12 ± 1.46 1,328	1–2 1–3 2–3	1.000 **0.017** 0.275
Change *n*	−0.41 ± 2.05 218	−0.42 ± 1.82 88	−0.22 ± 1.32 1,328	1–2 1–3 2–3	1.000 0.263 0.654
S-Urate, mmol/L					
Baseline *n*	334 ± 82 247	338 ± 77 95	340 ± 81 1,440	1–2 1–3 2–3	1.000 0.681 1.000
Follow-up *n*	318 ± 86 206	340 ± 83 86	329 ± 83 1,273	1–2 1–3 2–3	0.086 0.151 0.686
Change *n*	−14.0 ± 60.0 206	4.1 ± 55.4 86	−11.4 ± 54.8 1,273	1–2 1–3 2–3	**0.033** 1.000 **0.036**
S-GGT, mmol/L					
Baseline *n*	0.65 ± 0.67 232	0.58 ± 0.54 94	0.60 ± 0.82 1,356	1–2 1–3 2–3	1.000 1.000 1.000
Follow-up *n*	0.60 ± 0.75 203	0.70 ± 0.79 82	0.54 ± 0.76 1,275	1–2 1–3 2–3	0.967 0.696 0.161
Change *n*	−0.08 ± 0.39 193	0.12 ± 0.47 81	−0.08 ± 0.72 1,219	1–2 1–3 2–3	0.071 1.000 **0.032**
S-P, mmol/L					
Baseline *n*	1.04 ± 0.20 247	1.02 ± 0.17 95	1.01 ± 0.21 1,440	1–2 1–3 2–3	0.818 **0.040** 1.000
Follow-up *n*	1.08 ± 0.22 207	1.00 ± 0.17 81	1.02 ± 0.19 1,280	1–2 1–3 2–3	**0.004** **<0.001** 1.000
Change *n*	0.034 ± 0.25 207	−0.015 ± 0.17 81	0.014 ± 0.23 1,280	1–2 1–3 2–3	0.337 0.769 0.848
B-Glu, mmol/L					
Baseline *n*	6.58 ± 3.24 247	6.42 ± 3.29 95	6.42 ± 3.03 1,440	1–2 1–3 2–3	1.000 1.000 1.000
Follow-up *n*	6.81 ± 3.96 163	6.44 ± 2.64 76	6.38 ± 3.12 1,035	1–2 1–3 2–3	1.000 0.342 1.000
Change *n*	−0.01 ± 2.06 163	0.06 ± 1.47 76	−0.10 ± 2.29 1,035	1–2 1–3 2–3	1.000 1.000 1.000

Significance after correction for sex between smoking groups is presented in the right column. Data with bold numbers indicates statistical significance (*p* <0.05).

### Intervention program

Following recruitment, patients participated in a structured intervention program in large group settings. The patients were educated on health-related issues (e.g., meal planning, smoking cessation (SC), stress management, and body image) and encouraged to engage in health-promoting exercise (e.g., walking and swimming) (24). These theoretical and physical training sessions totaled 53 h over the 4-week period. In addition, in small groups, 5 h were spent on food evaluation (energy density and nutrient content of foods) and 5 h on cooking (*n* = 6–8 in each lesson). Patients were trained in how to make low-energy/low-fat and nutrient-dense dishes and were encouraged to consume whole wheat breads. In general, patients were encouraged to reduce fat intake and increase intake of vegetables, fruits, and fish. The food served at the center followed these recommendations and was calculated to have a high nutrient density. The average daily total energy intake was 6,300 kJ (1,500 kcal). Effects on CVD risk factors from changes in food selection over 1 year, corrected for weight loss, have been reported elsewhere (25). The study is registered as a sub-study of the Lifestyle Intervention Trial number: ISRCTN79355192.

### Laboratory analyses

At baseline and follow-up, laboratory analyses were conducted to assess metabolic risk factors. All patients had blood drawn in the fasting state in the morning before initiation of the intervention program. Measurements included the following: total serum cholesterol (S-Chol), triglycerides (S-TG), phosphate (S-P), urate (S-Urate), and gamma-glutamyl transferase (S-GGT). All blood samples were analyzed according to standardized routine procedures of the Department of Clinical Chemistry, University Hospital, Umeå, Sweden.

### Statistical analysis

Baseline characteristics of metabolic biomarkers from the blood samples was analyzed with Student’s *t*-test, and *p*-values were based on two-sided tests of statistical significance. A chi-squared test was used to analyze the difference between the proportions of patients within each group. Bivariate correlations were analyzed using Pearson’s correlation coefficient. Differences at baseline and follow-up, and changes over time between groups were tested for continuous variables using analysis of variance (ANOVA).

Less than 1% of the studied population did not attend the follow-up. Therefore, the number of patients included in the analysis was limited to those who provided blood samples at follow-up. The number of subjects (*n*) for each of the studied variables is included in the tables with group comparisons. Multiple linear regression (backward elimination) was performed with BMI change as the dependent variable. An interaction between changes in S-P and BMI, and between S-P and S-GGT, according to smoking habits, was constructed and included. Multiple logistic regression odds ratio [OR; 95% confidence level (CL)] for variables predicting risk with SC was performed to compare with risk in smoking and non-smoking subjects.

The program Predictive Analytics SoftWare (PASW Statistics, version 18.0.3 SPSS, Inc., Chicago, IL, United States) was used for the analysis.

## Results

At baseline, current and former smokers were younger and had lower SBP but higher S-TG than non-smokers (Table 1). Compared to non-smokers, smokers had lower DBP and higher S-P. There was no difference in BMI, S-Chol, S-Urate, S-GGT, or B-Glu between the three groups at baseline or at follow-up. At follow-up, current smokers had lower SBP but higher S-TG than non-smokers. Current smokers had higher S-P than non-smokers at baseline and higher than former smokers and non-smokers at follow-up.

Table 1 shows the changes in absolute terms for the studied variables in these three subgroups of smoking habits. A significantly less weight decrease was revealed in patients who quit smoking compared to patients who continued smoking or remained non-smokers. S-Urate increased with SC in comparison to a decrease in smokers and non-smokers. S-GGT increased with SC and, in contrast, decreased in non-smokers. The prevalence of decreased weight was 60% for SC, 82% for smoking, and 84% for non-smoking subjects.

Changes, expressed in percent, reveal less weight decrease in former smokers than in smokers (*p* = <0.001) and non-smokers (*p* = <0.001) and less increase in S-P than in smokers (*p* = 0.034) (Table 2). An increase in S-Urate and S-GGT was revealed in patients who quit smoking as compared to smokers (*p* = 0.024 and *p* = 0.046, respectively) and to non-smokers (*p* = 0.019 and *p* = 0.013, respectively).

**Table 2:** Percent change (%) in the metabolic risk variables in smokers who continue to smoke and in smokers who quit smoking during the 4-week intervention from baseline to first follow-up and in non-smokers at either the 6-month or 12-month follow-up.

Changes in %	Current smokers (1)	Former smokers (2)	Non-smokers (3)	Group test	*p*
BMI, kg/m^2^ *n*	−5.0 ± 6.5 241	−1.5 ± 6.0 92	−4.8 ± 5.6 1,414	1–2 1–3 2–3	**<0.001** 0.499 **<0.001**
SBP, mm Hg *N*	−3.7 ± 12.2 245	−2.3 ± 14.4 95	−4.40 ± 12.39 1,427	1–2 1–3 2–3	0.416 0.379 0.112
DBP, mm Hg *n*	−4.1 ± 12.8 244	−2.3 ± 14.7 95	−5.3 ± 13.0 1,414	1–2 1–3 2–3	0.273 0.157 **0.028**
S-Chol, mmol/L *n*	−0.88 ± 13.6 220	0.31 ± 19.7 88	−1.46 ± 14.51 1,347	1–2 1–3 2–3	0.546 0.581 0.280
S-TG, mmol/L *n*	−1.7 ± 41.6 218	−4.3 ± 49.3 88	−1.4 ± 44.4 1,328	1–2 1–3 2–3	0.664 0.922 0.554
S-Urate, mmol/L *n*	−3.0 ± 18.0 206	2.2 ± 17.1 86	−2.2 ± 16.7 1,273	1–2 1–3 2–3	**0.024** 0.523 **0.019**
S-GGT, mmol/L *n*	7.3 ± 53.0 193	32.5 ± 107.3 81	6.6 ± 89.8 1,216	1–2 1–3 2–3	**0.046** 0.925 **0.013**
S-P, mmol/L *n*	5.9 ± 25.0 207	0.30 ± 17.7 81	4.1 ± 23.2 1,280	1–2 1–3 2–3	**0.034** 0.289 0.155
B-Glu, mmol/L *n*	0.47 ± 21.34 163	2.79 ± 18.70 76	1.81 ± 31.5 1,035	1–2 1–3 2–3	0.418 0.598 0.790

Data with bold numbers indicates statistical significance (*p* <0.05).

Partial correlations revealed that percent changes in S-P correlated inversely with percent changes in BMI in former smokers (*r* = −0.358; *p* = <0.001) and non-smokers (*r* = −0.077; *p* = 0.006) but not in current smokers (*r* = −0.013; *p* = 0.858) (Table 3).

**Table 3:** Partial correlations (*r*, *p*-value) between percent change (%) in BMI and the metabolic risk variables in smokers who continue to smoke (current smokers) and in smokers who quit smoking (former smokers) and non-smokers, from baseline to first follow-up at either the 6- or 12-month follow-up.

Changes in %	Current smokers (1)	*p*	Former smokers (2)	*p*	Non-smokers (3)	*p*
SBP, mm Hg *n*	−0.002 235	0.973	0.101 88	0.341	**0.062**	**0.021**
DBP, mm Hg *n*	−0.011 234	0.870	0.152 88	0.153	**0.053** **1,385**	**0.048**
S-Chol, mmol/L *n*	**0.215** **215**	**<0.001**	0.115 83	0.293	**0.180** **1,332**	**<0.001**
S-TG, mmol/L *n*	**0.245** **213**	**<0.001**	0.156 83	0.155	**0.270** **1,313**	**<0.001**
S-Urate, mmol/L *n*	**0.186** **201**	**0.008**	**0.254** **81**	**0.020**	**0.225** **1,258**	**<0.001**
S-GGT, mmol/L *n*	**0.162** **188**	**0.025**	0.192 76	0.092	**0.091** **1,201**	**0.002**
S-P, mmol/L *n*	−0.011 202	0.876	**−0.358** **76**	**<0.001**	**−0.077** **1,266**	**0.006**
B-Glu, mmol/L *n*	**0.198** **159**	**0.012**	0.210 71	0.075	**0.110** **1,022**	**<0.001**

Each variable was adjusted for sex and age. Data with bold numbers indicates statistical significance (*p* <0.05).

Table 4 shows the results from multiple linear regressions, with percent change in BMI as the dependent variable. An association was observed between changes in BMI (%) and in S-P (%) among former smokers. Change in BMI (%) was associated with change in S-GGT in non-smokers and with change in S-Urate in smokers.

**Table 4:** Multiple linear regression with change in BMI (%) as the dependent variable.

Variables	Current smokers (1)		Former smokers (2)		Non-smokers (3)	
	B-value	*p*	B-value	*p*	B-value	*p*
Age	0.079	0.101	0.046	0.525	0.003	0.877
T2DM	1.718	0.171	0.534	0.755	**1.751**	**<0.001**
Sex	−0.150	0.885	0.352	0.837	0.645	0.074
Baseline						
BMI, kg/m^2^	**−0.176**	**0.046**	−0.225	0.166	**−0.214**	**<0.001**
S-Urate, mmol/L	−0.006	0.403	−0.023	0.051	**0.006**	**0.012**
S-GGT, mmol/L	−0.354	0.644	1.844	0.208	0.097	0.601
S-P, mmol/L	4.385	0.094	−8.116	0.146	0.098	0.917
Change, %						
S-Urate	**0.055**	**0.040**	0.042	0.324	0.075	0.055
S-GGT	0.014	0.119	0.007	0.054	**0.004**	**<0.001**
S-P	−0.009	0.675	**0.052**	**0.005**	−0.014	0.089
*R* ^2^	**0.150**	**<0.001**	**0.322**	**0.004**	**0.105**	**<0.001**

Data with bold numbers indicates statistical significance (*p* <0.05).

Results from multiple logistic regression, with former smoker (=1) and smoker (=0) as dependent variables, are given in four models (Table 5). Interactions between S-P and S-GGT (S-P*SGGT) and between S-P and BMI (S-P*BMI) were included if significant. These interactions were independently associated with smoking habits. Adding both interactions in the regression indicates that smokers, but not former smokers, had an association with S-P*BMI. Results from multiple logistic regression with former smoker (=1) and smoker (=0); former smoker (= 1) and non-smoker (= 0); and smokers (=1) and non-smokers (=0); as dependent variables are given in Table 6. Former smokers had an increased risk for weight gain compared to smokers and non-smokers. An increase in S-GGT could be seen in former smokers compared with smokers; conversely, S-P was increased in smokers.

**Table 5:** Multiple logistic regression with odds ratio (OR) and *p*-value for variables predicting risk with smoking cessation (SC) = 1 compared to being a smoker and continuing smoking at follow-up (SM) = 0, not included in the equation = −----.

Former vs. current smokers								
Variables	OR	*p*	OR	*p*	OR	*p*	OR	*p*
T2DM	0.997	0.994	0.917	0.823	1.003	0.994	0.925	0.841
Sex	1.335	0.389	1.270	0.484	1.269	0.486	1.244	0.527
Baseline								
BMI, kg/m^2^	0.983	0.560	0.980	0.511	0.988	0.691	0.984	0.599
S-P, mmol/L	0.057	**0.004**	0.065	**0.006**	0.054	**0.003**	0.061	**0.005**
S-GGT, mmol/L	1.248	0.466	1.271	0.469	1,278	0.432	1.309	0.425
Changes, %								
BMI	1.338	**0.003**	1.305	**0.006**	1.425	**<0.001**	1.386	**0.002**
S-P	0.118	**0.017**	0.133	**0.024**	0.063	**0.005**	0.072	**0.008**
S-GGT	3.698	**0.008**	7.530	**0.002**	3.496	**0.009**	6.787	**0.003**
S-P * S-GGT	-----	-----	0.002	**0.026**	-----	**-----**	0.004	0.060
S-P * BMI	-----	-----	-----	-----	0.324	**0.019**	0.376	**0.046**
*Nagelkerke* *R^2^*	*0.184*		*0.210*		*0.211*		*0.229*	

T2DM, type-2 diabetes mellitus; BMI, body mass index, kg/m^2^; S-P*S-GGT, interaction S-P and S-GGT; S-P*BMI, interaction S-P and BMI. Data with bold numbers indicates statistical significance (*p* <0.05).

**Table 6:** Multiple logistic regression OR (95%CL) for variables predicting risk with smoking cessation (SC) = 1 compared to being a smoker and continuing smoking at follow-up SM = 0; smoking cessation (SC) = 1 compared to being a non-smoker at follow-up SM = 0; smoking = 1 compared to being a non-smoker at follow-up = 0.

Variables	Former vs. current smokers		Former vs. non-smokers		Current vs. non-smokers	
	OR	*p*	OR	*p*	OR	*p*
T2DM	1.003	0.994	0.899	0.734	0.973	0.894
Sex	1.269	0.486	0.908	0.710	0.816	0.234
Baseline						
BMI, kg/m^2^	0.988	0.691	0.991	0.709	1.001	0.945
S-P, mmol/L	0.054	**0.003**	0.596	0.518	6.715	**<0.001**
S-GGT, mmol/L	1.278	0.432	0.956	0.794	1.001	0.995
Changes, %						
BMI	1.425	**<0.001**	1.435	**<0.001**	0.969	0.474
S-P	0.063	**0.005**	0.345	0.163	3.072	**0.011**
S-GGT	3.496	**0.009**	1.410	0.092	1.009	0.946
S-P * BMI	0.324	**0.019**	0.517	**0.042**	0.959	0.790
*Nagelkerke R* ^2^	*0.211*		*0.077*		*0.022*	

Data with bold numbers indicates statistical significance (*p* <0.05).

## Discussion

The 4-week comprehensive lifestyle intervention resulted in weight reduction in 60% of patients who quit smoking, as compared to 82% of patients who continued smoking and 84% of those who were non-smokers at baseline and at follow-up. Many studies, including meta-analyses, report both weight gain and weight loss, indicating the complexity of the underlying metabolic effects associated with SC (26). Aubin et al. (26) report that up to 21% of participants lost weight, compared to 48% who gained weight, at 1 year post-cessation. A cohort study of an SC program in Brazil showed weight maintenance or an increase of not more than 5 kg over 12 months in 65% of the participants, whereas 11% gained more than 10% of their body weight (27). Predictive factors for weight loss, as opposed to large weight gain after SC over both short and long time, need to be further elucidated (3).

The high prevalence of weight loss in the present study, rather than weight gain, allows studies on metabolic consequences independent of weight gain. Changes in body weight were calculated from BMI change and in percent from baseline to follow-up in a patient population at risk from obesity related disturbances. It is important to study weight change as a percentage rather than in absolute amount in kilos, to exclude bias from differences in weight at baseline. The multiple linear regressions included interactions to support findings from metabolic differences between the subgroups studied, in addition to changes in body weight. We hypothesize that the impact of SC on metabolic disturbances is associated with interactions between P-Phos, P-Urate, and P-GGT.

The highly structured inpatient program for dietary pattern and physical activities at the VPE-center included individual guidance during the course at the center and advice for home management (24). The overall aim of the intervention was to decrease CVD risk by reducing body weight and to limit weight gain with SC (22). It remains to be elucidated whether there is any specific diet that can limit weight gain in connection with smoking cessation. For example, a higher ratio of carbohydrate/protein in the diet is associated with metabolic changes and weight gain in SC (28). However, it remains unclear whether a lower ratio will limit weight gain. Improved understanding of the physiological mechanisms underlying SC can lead to more accurate prediction and control of weight change during lifestyle interventions. Thus, enabling improved dietary guidelines focused on limiting weight gain. In the current patient population, altered food selection resulting in weight change affected CVD risk factors (25).

A high rather than low BMI is predictive of weight loss during and after SC intervention (29). However, over 8 years, obese abstainers gained more weight compared to smokers (30), and in another obese population, 1 year after SC, weight change was similar for smokers and non-smokers (31). Therefore, we suggest that part of the convincing outcomes in the present study were a result of the changed habits following the comprehensive program, and to some extent, the high BMI at baseline.

Assessing the benefits from SC in relation to changes in vascular risk factors and weight change necessitates a focus on both baseline levels and specific metabolic alterations. In addition to weight loss, former smokers had a decrease in S-TG, S-Chol, SBP, and DBP. Moreover, an increase in S-Urate and S-GGT was observed compared with both smokers and non-smokers. These results indicate specific metabolic effects from SC. In addition, S-P did not increase as much as expected compared to smokers and non-smokers in relation to weight reduction. The correlation between BMI change in percentage and change in S-P% was highly significant among former smokers but not among smokers. These results support involvement of a metabolic effect that reflects weight regulation.

The degree of change, however, differed across the three smoking subgroups. Former smokers had less of a decrease in the SBP, and the decrease in S-TG was almost double that of non-smokers, which might be associated with their higher baseline level. Both weight loss and physical activity, in addition to SC, can contribute to a decrease in S-TG. It has been shown that weight gain following SC is associated with an increase in S-TG and a decrease in SBP (9, 10). May the higher level of S-TG at follow-up for former smokers, compared to non-smokers, represent the relatively high level at baseline, that is, do subjects who quit smoking reach a comparable level of S-TG as non-smokers at follow-up in relation to their initial levels? Previous studies have shown associations between high levels of S-TG with smoking and changes in weight (32). It has been reported that smokers increase P-TG in addition to an increase in resting metabolic rate, together with a lowered energy intake and no gain in weight (33).

Furthermore, the effect of weight change (i.e., gain or loss associated with SC) on changes in blood pressure, amount of smoking, and body weight at baseline also needs to be further elucidated. Smokers increase their P-TG with an increase in both resting metabolic rate and energy expenditure, along with a lowered energy intake and no gain in weight (33). Changes in both DBP and S-TG concentrations were shown to be more favorable in obese quitters after 12 weeks of a dietary intervention (32). This finding indicates that smoking *per se*, with its high S-TG, contributes to the increased CVD risk. If a change in a CVD risk factor does not follow a weight change in an expected manner, metabolic disturbances due to smoking habits may be involved. We need more knowledge about the mechanisms involved in the regulation of energy metabolism with SC. Results from the present study support a role for plasma phosphate levels, possibly due to their effect on intracellular energy metabolism.

The large discrepancies between results on weight changes from different studies may depend on metabolic differences before SC that influence weight gain. Smokers most often weigh less than non-smokers, maybe due to a difference in energy intake. Moreover, the gain in weight upon SC could be a consequence of consuming more food. However, smokers have been shown to consume more energy per day than non-smokers and weigh less (34). Patients who quit smoking lost less body weight than continuous smokers and non-smokers in the present study, indicating a difference in energy metabolism.

Analysis of smoking status in relation to phosphate levels revealed that smokers with high levels of S-P and non-smokers with low levels of S-P at baseline had an increase in S-P associated with weight loss. A decrease in S-P levels was associated with SC (in contrast to an increase in smokers and non-smokers) in relation to weight loss. The negative association between the change in S-P and BMI (%) with SC might explain the observed weight gain. By testing for interactions between S-P and smoking on the change in BMI (%) in the logistic regression, we found that a decrease in S-P resulted in weight gain and a conversely increase in S-P in weight loss. These results strengthen the theory that S-P can be used as a marker for weight change. The weight loss was larger with an increase in S-P in smokers and non-smokers, while less with a decrease in S-P in former smokers. It has been shown that low S-P is associated with decreased metabolic rate (35), high BMI (23, 36), adiposity (37), and metabolic syndrome (23, 38). In the present study, the decrease or less increase in S-P together with an increase in S-Urate and S-GGT are specific for a disturbed metabolism in relation to smoking cessation.

A rapid change in energy balance has been observed closely after the time of SC, which is reversed on resumption of smoking (39). This effect depends on the years and the amount of smoking before SC (40). Smoking might be associated with a set point in the metabolism, with secondary changes in eating (41). We hypothesize that there is an increased demand for phosphate to generate adenosine triphosphate (ATP) after SC, which can explain why S-P decreases rather than increases in individuals who lost weight after SC.

As smoking disturbs factors I, III, and IV in the electron transport chain in mitochondrial cells, smokers have lower ATP production than non-smokers (40, 42). Disturbed ATP generation and decreased resting metabolic rate are associated with low levels of S-P (35). Additional support for the phosphate hypothesis is that weight loss is improved in adults with obesity by phosphate supplementation (43) and that K-Mg-Phosphate, added to glucose, gives rise to higher postprandial thermogenesis in obese but not lean females (44).

A weakness of this study concerns the grouping of patients who were admitted to follow-up, either at 6 months or 12 months. Information on the individual amount of smoking before quitting, dietary energy intake, and level of physical activity during follow-up is lacking. A strength of this study is that it evaluates a common comprehensive program with a focus on lifestyle habits, and that individual advice for lower and controlled dietary intake, higher activity level, and stress control was supported by professional staff at the center. A high level of motivation to lose weight, together with teamwork in achieving better health, corresponds to the aim of this intervention.

In conclusion, the 4-week intervention—with decreased energy intake and daily physical activity, focused on weight reduction—resulted in weight loss in patients who quit smoking. In addition, a decrease in S-P in connection with SC was associated with weight gain, compared to an increase in S-P with weight loss. The results of the present study support the conclusion that changes in S-P levels with SC are associated with the degree of weight change. Future research on metabolic risk with both intentional and unintentional gain or loss of body weight needs to consider the level of phosphate as an important nutritional biomarker associated with health and disease.

## Data Availability

The datasets presented in this article are not readily available because data cannot be shared publicly because of ethical and legal reasons, since public availability would compromise patient confidentiality. Data are available on request, if approval from the Regional Ethics Committee is given. Requests may be sent to the corresponding author or to the Regional Ethics Committee, Umeå. Requests to access the datasets should be directed to Regionala Etikprövningsnämnden i Umeå, Samverkanshuset, C/O Umeå Universitet, 901 87 UMEÅ SWEDEN E-mail: epn@adm.umu.se.
